# Enhancement the performance of swirl heat exchanger by using vortices and NanoAluminume

**DOI:** 10.1016/j.heliyon.2019.e02268

**Published:** 2019-08-27

**Authors:** Walaa M. Hashim, Hisham A. Hoshi, Huda A. Al-Salihi

**Affiliations:** Department of Electromechanical Engineering, University of Technology, Baghdad, Iraq

**Keywords:** Mechanical engineering, Nanotechnology, Heat exchanger, NanoAluminume, Swirl, Vortices

## Abstract

Heat exchangers are widely used in many industrial applications. It is well known that the effectiveness of these exchangers is normally measured by the amount of the heat transfer, which should be minimized as much as possible according to their size. Present study pays more attention to examine Taylor vortex which formed in the flow of the hot water of the smooth surface heat exchanger and a swirl flow of cold air. Also, an attempt was made to improve the result by replacing the heat transfer surface by Nanoaluminume metal. Experimental results revealed that the effectiveness of the exchanger at the ratio (C_min_/C_max_ = 0.08) could be enhanced the effectiveness of the standard exchanger at the ratio (C_min_/C_max_ = 0). Further, it was found that using nano Aluminume as a medium material for heat transfer increased the effectiveness (3.2%, 4.3% and 4.58%) for a rotational speed of inner cylinder which were used (0, 65and 80) rpm, respectively.

## 1 Introduction

It is generally accepted that one ways to increase the amount of the heat transfer through heat exchangers is generating vortices. In fact, vortices tend to increase the surface area of the heat transfer. Meanwhile, these vortices decrease the effects of the boundary layer. That could lead to a sharp increase in the amount of heat transfer through the exchange [1, 2]. The finite swirl flow is another way to increase the time required for a fluid to heat transfer exchanging [3, 4, 5, 6] as well as using high conductivity heat transfer medium.

F. Chang and V. Dhir [7], observed the turbulent flow field in the tube which was regularly heated from the wall, when the fluid injected tangentially. The tube was injected by the air through the injectors which designed as 88.9-mm inner diameter and 2.5-m long acrylic tube. Six injectors with 22.23-mm inner diameter were successfully used. The experimental results identified the two major mechanisms of heat transfer enhancement. first in high axial velocity near the wall to increase the wall heat flux and at high turbulence level improved the mixing in which lead to increased heat transfer.

Wan and Coney [8], focused on the narrow and wide gaps at radius ratios N = 0.955 and N = 0.8. The characteristics of the heat transfer and the modes of transition were observed together. The onset of vortex flow and its higher transitions revealed a significant rise in Nusselt number. Further, an obvious change in the Nusselt number occurred at higher Taylor numbers, of the order of 10^6^, as the onset of the transition to periodic turbulent vortex flow.

H.Jagdale et.al [9], examined the effect of heat transfer enhancement of swirl generator with tangential entry of fluid. Six tangential entry nozzles were placed such that they were equidistance along the length of pipe with water as the working fluid, cold water flowing through annulus space to generate swirling motion. It was observed that the heat transfer rate increased with Reynolds number. The maximum heat transfer rate increased about 67 % with tangential entry fluid than the heat exchanger without a tangential entry.

M. Lopez et.al [10], studied the heat transfer of fluid flow among a heated rotating cylinder with a cooled fixed cylinder. Heat transfer in the laminar regime occurred only through conduction as in a solid by assuming the cylinders as infinite length. The influence of the geometric parameters was comprehensively studied by varying the radius ratio and length-to-gap aspect ratio with a wide range of Prandtl, Rayleigh, and Reynolds numbers. It was obtained a simple criterion, Ra. a(ɳ)< Ґ. It was found that there was no effect of Prandtl number on the coefficient. However it was strong with respect to Reynolds number even outside the laminar regime.

K.Saravanan and R. Rajavel [11], inspected the heat transfer coefficients of benzene in a spiral heat exchanger. The tests were carried out by examining several variable such as the mass flow rate, temperature and pressure of cold working fluid. Reasonable comparison was made between the experimental and theoretical results. A new relationship for the Nusselt number which can be used for many applications was also suggested.

A. Aubert et.al [12], investigated the heat with mass transfer of the rotor-stator cavity of an electrical motor modeled by a Taylor-Poiseuille scheme with an axial through flow. The values of the Nusselt number were measured to be related to the rotor boundary layer thickness to the power −1/10. The features of decaying swirling flows and forced convective heat transfer to the state of turbulent flow in a fine concentric annulus were simulated by A. Jawarneh [13]. The main equations were numerically solved by a finite volume method. The uniform wall temperature in the interior and the adiabatic wall of the external wall were considered as thermal boundary conditions. Simulation results were found at different values of the inlet swirl number and the Reynolds number. Simulations appearance that the inlet swirl number had excessive effects on the heat transfer characteristics.

J.Khorshidi and S. Heidari [14], also simulated the performance and applications of a spiral heat Exchanger. The major equation of the heat transfer phenomena in such heat exchangers was unfavorably discussed. Regarding the main equations a LAB-sized model of this type of heat exchanger was designed and built Nusselt number increases as them mass flow rate increases. The average Nusselt number is about 100 that is very good. Further, D. Liu et.al [15], studied Taylor vortex flow, which was extremely affected by a constant temperature that drop in the plain and slot wall models. Experimental and numerical results were successfully conducted by a reasonable comparison between them. It was found that the heat flux had a maximum value in the 12-slot prototypical, and this prototype had the best heat transfer capacity.

The transient heat transfer through the aluminum alloy ship deck panels under application of the local heat transfer similar to that of a vertical take-off and landing aircraft exhaust plume core in typical operation was inspected by K. Crosser [16]. It was reported that the heat transfer was more dependent on the flow conditions than the variations in the geometry of the deck panels owing to the low variation in thermal resistance across the plate.

R.Aghayari [17] on the other hand examined the heat transfer coefficient of nanofluids in double tube heat exchanger with turbulent flow and the relevant effective parameters. The experimental results showed a significant 8%–10% rise in the overall heat transfer coefficient. Accordingly, with an increase in the processing temperature and/or particle concentration the overall heat transfer coefficient was observed to increase.

V. Zarko [18], investigated the ignition and combustion of nano Al particles in conditions of a shock tube and in a plastic tube. Some peculiar properties of heat transfer between metal nanoparticles and gaseous environment in a shock tube and in a plastic burn tube were critically examined. It was found that the nano particles at high temperatures and pressures were enhanced to be thermally isolated from the ambient gas environment. Experimental results by Wan and Coney [19] and Kosterin and Finatev [20], showed that Taylor vortex flow exhibit high efficient flow. While, Sparrow and Chaboki [21] and Clayton and Morsi [22], confirmed that the swirl could lead to the considerable increase in the heat transfer rate.

From the above previous studies, it seems that there was no attempt has been made to improve the heat transfer by using more than one method simultaneously. Accordingly, present study emphasized to enhance the performance of heat exchanger by using three experimental methods, as explained below:

1. Generate Taylor vortices in the inner annular in specific between the inner and middle cylinder by the rotation of the inner cylinder at a reasonable rotational speed which was previously determined.
2. Generate of swirl flow in the outer annular through the slots that exist on the surface of the cylinder external.
3. Use a proper nanomatrials with a high thermal conductivity such as nano aluminum -powder which was successfully used as a heat transfer surface between hot fluid (water) and cold fluid (air).

In fact, the enhancement of the performance of heat transfer by correlating these three methods considered to be a choice to enhance the heat transfer than using fins in the heat exchanger.

## 2 Methodology

**Fig. 1:**
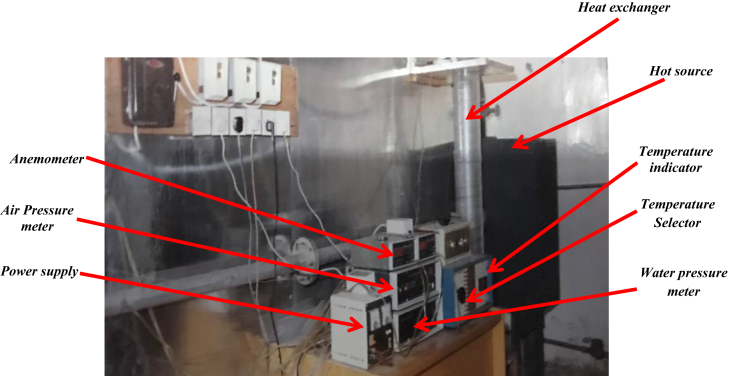
Experimental apparatus.

**Fig. 2:**
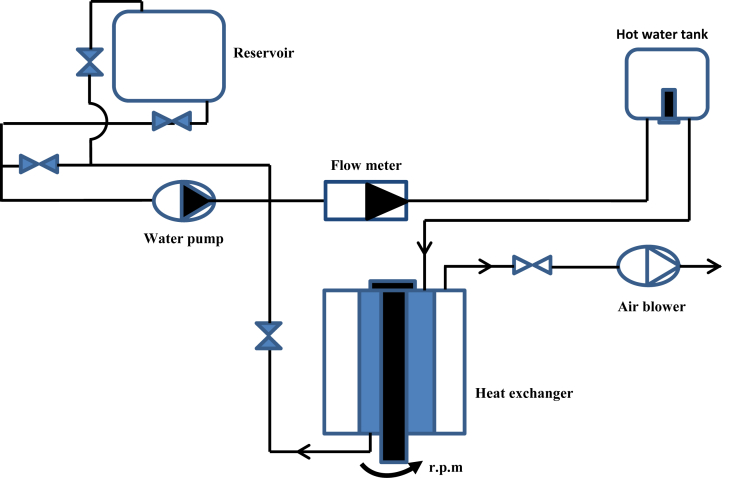
Schematic diagram of experimental apparatus.

The experimental apparatus is shown in Figs. 1 and 2 which involve three concentric cylinders with annular heat exchanger. The middle and the outer cylinders were effectively fixed, while the inner cylinders were designed to be moved and run by a belt connected to a variable-speed D. C motor have 0.3 h. p. The external diameter of the inner, middle and outer cylinders were designed as 0.0593m, 0.0893m and 0.185m, respectively. Whereas, the internal diameters of the middle and outer cylinder are 0.0838m, and 0.178m, respectively. All cylinders had the same length 1.25m which represents the heat exchanger length.

The radius ratio (outer inner cylinder radius to inner middle cylinder radius) of the internal annular space was used N = 0.8 to generate vortices in hot water flow. Inner cylinder was made from Aluminum as a hollow shaft with smooth surface in order to reduce the horsepower that required for cylinder rotation. To get a high conductivity for the heat transfer, the middle cylinder was made from bulk Aluminum once and another one from nanoaluminum.

**Fig. 3:**
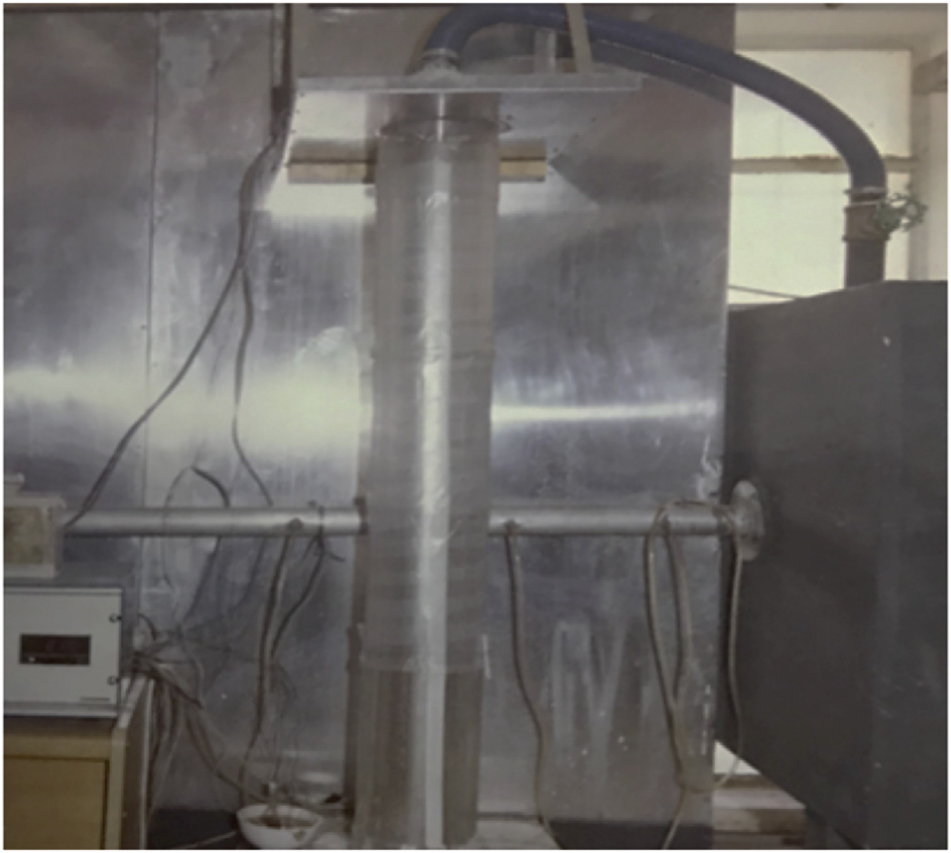
Heat exchanger.

**Fig. 4:**
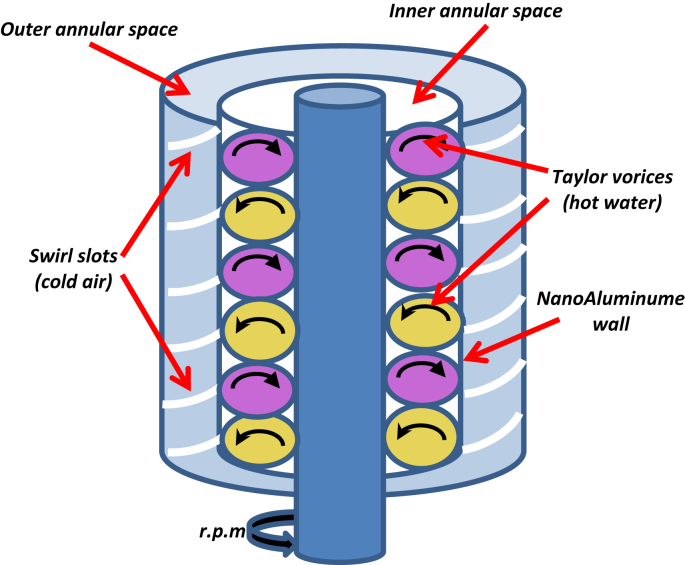
Heat exchanger cross section.

To obtain a swirl flow in the outer annular space (between the external diameter of middle cylinder and inner diameter of outer cylinder), outer cylinders contain longitudinal slots with a wide 1.5 mm which were distributed uniformly around a cylinder circumference. It should be applied through the wall of the cylinder, which was relatively thick so that it was touching the inner surface of the cylinder in order to make it suitable for the generation of the required swirl during the outer annular space between the outer and middle cylinders when the fluid passes through the space as shown in Figs. 3 and 4.

In order to reduce the thermal losses from the heat exchanger, the outer cylinder was made from adiabatic perspex metal. The hot water was heated up by using an electrical heater with 6000 W. In order to reduce pipe heat losses and reach the steady state as much as possible, hot water was passed through the isolated pipe network with diameter 12.5 mm. In addition, hot water was circulated in the system by a centrifugal pump with a capacity of 0.5 h. p.

A blower 10 h. p on the other hand, was used to generate a swirl flow in outer annular space. To obtain a variable hot water and cold air flow rate ball valve was used in both conduits. The hot water flow rate was measured by using a ball flow meter while for cold air a digital vane anemometer was used. To measure the inlet and outlet temperature for both water and air a thermocouple type k was used with a digital thermometer. A laser tachometer was used to measure the rotational speed of the inner cylinder.

### 2.1 Nanomaterial

Present study utilized nanosized of aluminum powders which critically prepared by a wire electrical explosion (WEE) in the High Voltage Research, since WEE is well known as an active technique for fabricating acceptable and reactive powders. In fact, the measurements were carried out by using Aluminium as bulk metal and aluminum as nanopowder material, in order to determine the role of the bulk density of the studied samples.

Prior experimental studies of nanofluids which involve metallic particles were utilized low size fractions of naoparticles. As a result, any size of the nanofluid that depends on the thermal conductivity was not seeming from these sizes. Accordingly, the data could be correlated using the bulk thermal conductivities of the solid and base fluid.

Three sizes of nanoparticles were examined to measure the thermal conductivity of nanofluids which covering different volume fractions of aluminum powder.

Literature data for nanofluids which containing sufficient metallic nanoparticles were compiled and fitted using Eq. (1) with and without considering the size dependence of the thermal conductivity of the particles [23].

(1) (k_eff_) n = k_p_ n ϕ + (k_l_) n (1 − ϕ) − 1 < n < 1

### 2.2 Calculation

The analysis can be presented as below which assumed that:

1. Energy loss to the atmosphere was neglected.
2. A steady-state conditions in heat exchanger.
3. Single phase in the fluids.
4. Heat capacities are constant.
5. The overall heat transfer coefficient is constant.

The following equations were used to calculate the number of thermal transfer units and longitudinal Reynolds number of heat exchanger for both water and air during the exchanger [24].

(2) $$\mathrm{NTU}=\frac{\mathrm{AU}}{C_{\min}}=\frac{q}{\mathrm{LMTD}.C_{\min}}$$

(3) $$q=m_{w}.Cp_{w}.\Delta T_{w}=m_{A}.Cp_{A}.\Delta T_{A}$$

(4) $$\mathrm{LMTD}=\frac{\Delta T_{1}-\Delta T_{2}}{\ln\frac{\Delta T_{1}}{\Delta T_{2}}}$$

(5) $$Re_{w}=\frac{u_{w}\left(2C_{1}\right)}{\vartheta_{w}}$$

(6) $$Re_{A}=\frac{u_{A}\left(2C_{2}\right)}{\vartheta_{A}}$$

(7) $$\varepsilon =\frac{m_{A}Cp_{A}\left(T_{A2}-T_{A1}\right)}{C_{\min}\left(T_{w1}-T_{A1}\right)}=\frac{T_{A2}-T_{A1}}{T_{w1}-T_{A1}}$$

Taylor's number of water can be determined from the outer radius of the inner cylinder and the inner diameter of the middle cylinder and the angular velocity of the inner cylinder [25, 26, 27], as expressed in Eq. (8).

(8) $$T_{a}=\frac{2\omega_{1}^{2}R_{1}^{2}C_{1}^{3}}{\left(R_{1}+R_{2}\right)\vartheta_{w}^{2}}$$

## 3 Results and discussion

In the present study it was utilized Taylor vortex flow of water in a hot water and the air swirl flow of cold fluid inside counter heat exchanger designed for this purpose. It was generating Taylor vortices by hot water at 80 °C inside annular space between overlapping cylinder, internal cylinder rotates while the other fixed. For cold air at room temperature swirl flow generated by a slot shape in the outer annular in which, surrounded first annular and separated by high conductivity metal (bulk aluminum & nanoparticles) in which represent a wall of heat transfer between Taylor vortex swirl flow measured at variable flow rates, various rotational speed of the internal cylinder and variable length of the slots in which swirl generating. It should be noted that the slot length of the generated swirl was indicated the true length of the uniform swirl flow.

To evaluation heat exchanger performance under study, inlet and outlet temperature of water and air was measured, which the vortex begins to decay towards the exit of the heat exchanger. Consequently, the effectiveness of heat exchanger (ε) and the number of heat transfer units (NTU) were successfully calculated.

**Fig. 5:**
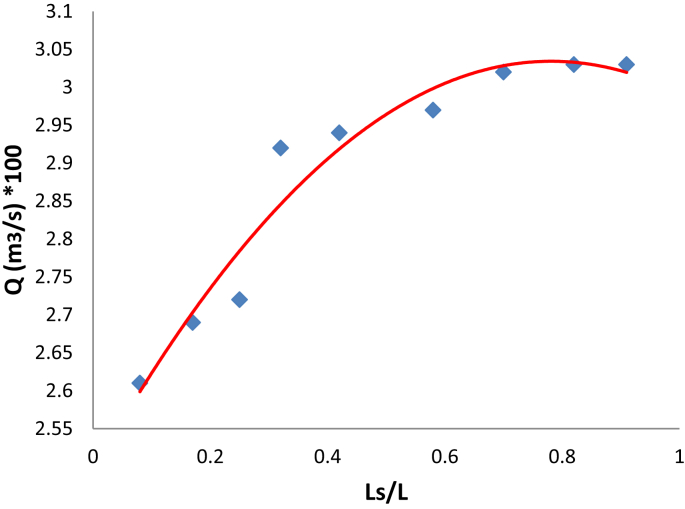
Variation of air flow rate with slots length.

Fig.(5) illustrates the change in the flow rate of air intake to the heat exchanger with the relative length of the slots that generated the vortices. It is obviously seen that the relative length of (0.468) allows the amount of external air to pass (0.03 m^3^/s) in which represent the maximum amount of external air about (0.91), which withdrawn from through the slots at its maximum length.

**Fig. 6:**
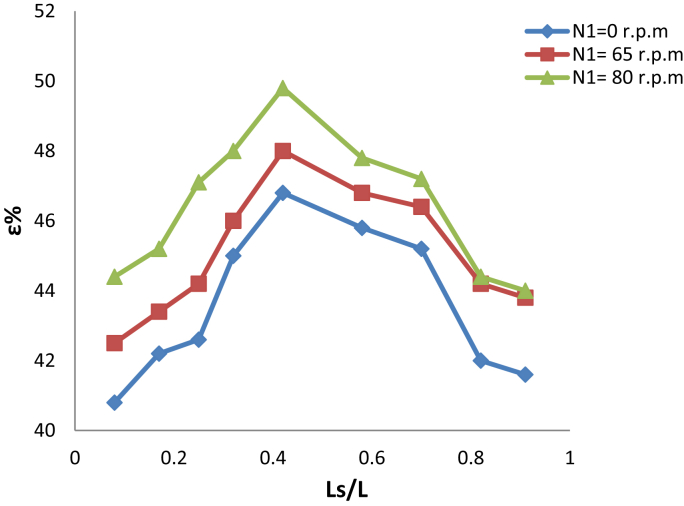
Variation of effeteness with slots length for bulk aluminum as wall.

Fig.(6) displays the change of the effectiveness of heat exchanger with the relative length of the slots by using a bulk aluminum as a medium heat transfer wall for three inner cylinder speed used (0, 65, 80) r.p.m. It's clear the effectiveness of heat exchanger was reached a maximum value 46.8%, 48% and 49,8% of relative slot length 0.42 at inner cylinder speed (0, 65 and 80) rpm, for the hot fluid flow rate 1.023 m^3^/s, which means different values for the Taylor number. It is clearly noticed that the maximum value of the effectiveness increases significantly as the internal speed of the cylinder increase. The increase in the length of the slots that generating a vortex over this length, which can be called effective length, means increasing in the length of the regular vortex and shortening the distance of the decomposition of the spiral during the heat exchanger. This result could be considered a positive on the hand and negative on the other hand. There is a part of the fluid not to take sufficient time and the surface area of the heat exchange with the other liquid. This is why the heat exchanger is less affected by increasing the length of the slots, producing the vortices after the effective length, which gives the maximum value of the effectiveness for the heat exchanger.

**Fig. 7:**
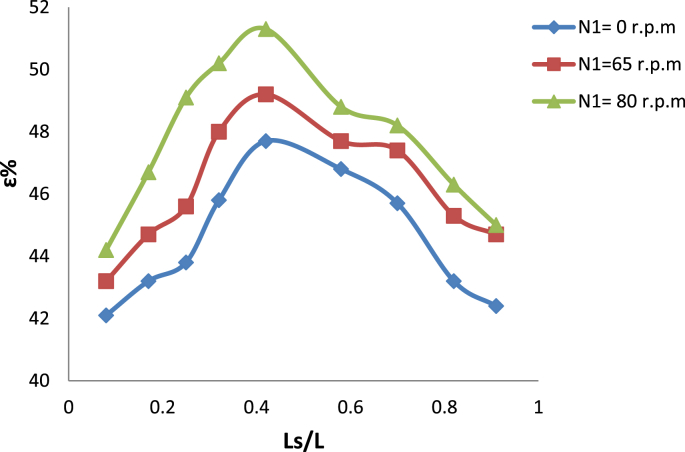
Variation of effeteness with slots length for nanoaluminum as wall.

Fig. (7) illustrates the effect of using nonaluminum instead of bulk aluminum as a medium of heat transfer between Taylor vortices in hot fluid (water) and swirl flow of the cooled fluid (air). Its note the increment in the effectiveness of the heat exchanger due to increase the conductivity of wall metal. The high effectiveness was recorded for three inner cylinder speed used (0, 65 and 80) r,p,m are 47.7%, 49.2% and 51.3% respectively. In order to determine the validity of the exchanger, it was compared the exchanger that under examination with the standard heat exchanger designed according to the international standards.

**Fig. 8:**
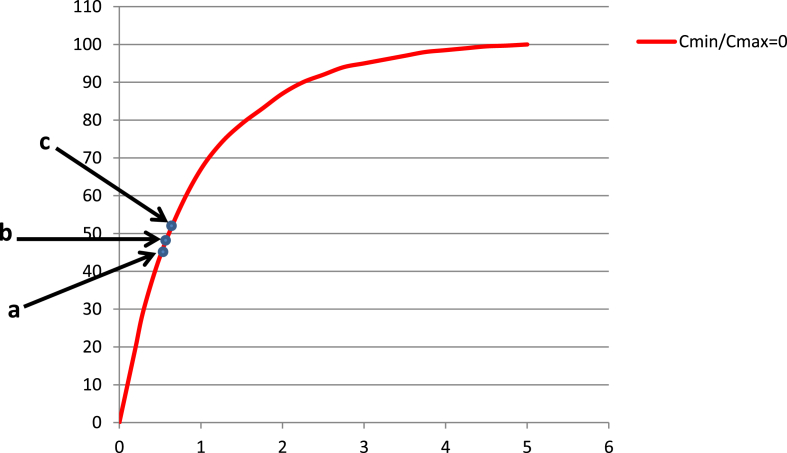
Performance of the standard exchanger.

Fig. (8) shows the performance of the standard exchanger with the counter linear flow of fluids. It is obvious to see that the effectiveness of the heat exchanger decreased significantly to increase the ratio of the minimum thermal content to the maximum of the heat exchange fluids (C_min_/C_max_). The points (a, b, c) represents the performance of the exchanger that examined at the ratio (C_min_/C_max_) for three speeds of the internal cylinder, at the effective length of the vortices generator (0, 65, 80) r.p.m, respectively. At the first speed (0 r.p.m), the flow between the middle and inner cylinders was a laminar flow, type couette flow. The flow at the second speed (65 r.p.m) was observed as Taylor vortices which was relatively stable. The vortices state at the third speed (80 r.p.m) was extremely changed to be unstable or wavy.

It can be seen from Fig. (8) the points that shown in Table 1 which are located on the performance curve at the ratio (C_min_/C_max_). This indicates that the effectiveness of the exchanger at the ratio (C_min_/C_max_ = 0.08) could be enhanced the effectiveness of the standard exchanger at the ratio (C_min_/C_max_ = 0). The superiority of the performance of current heat exchanger compared with the slandered counter heat exchanger considered to be the best criterion to reach the optimum results for using the vortices and nanomaterials in the heat exchange of the fluids, in order to reduce the surface area of heat exchange as well as increase thermal conductivity of heat transfer metal. It is well known that the vortices seek to increase the rate of heat transfer of the unit area as a result of the decay of the thermal boundary layer by mixing vortices.

**Table 1:** Points property.

*Point*	*Re*_*w*_	*Re*_*a*_	*N (r.p.m)*	*Ta*	*NTU*	*ɛ%*
a	2135	8760	0	0	0.641	47
b	2135	8760	65	1.162E4	0.6685	48
c	2135	8760	80	1.76E4	0.7086	50

**Fig. 9:**
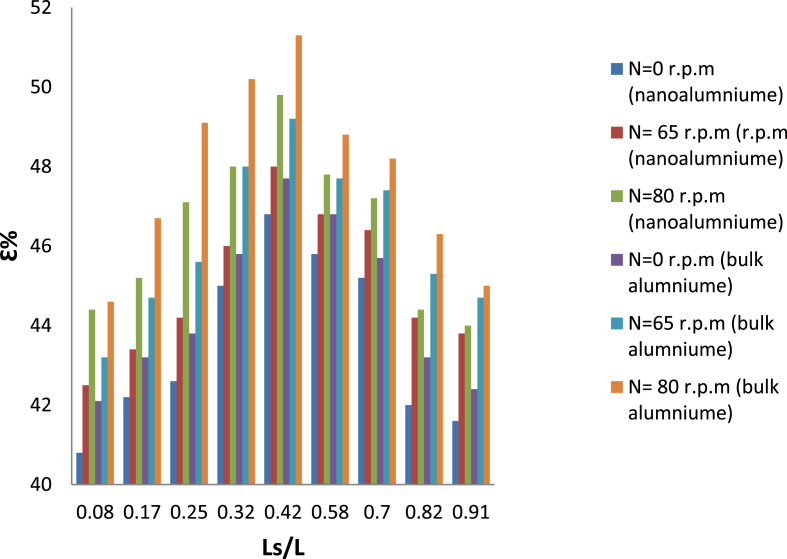
Variation of effectiveness with bulk and nonoaluminum wall.

Fig. (9) shows a comparison between the effectiveness of current heat exchanger by using bulk aluminum once and nanoaluminum once again as wall heat transfer medium. It's clear enhance the of heat exchanger by using nanoaluminum due to increase the thermal conductivity of the metal. 3.2%, 4.3% and 4.58% represent the maximum increment for a rotational speed of inner cylinder are used (0, 65, and 80) rpm, respectively.

## 4 Conclusions

The most important conclusion to be drawn from the current study is the possibility of introducing a new type of heat exchanger that exceeds its performance on the standard heat exchanger by adopting vortices in the heat exchange between the fluids and aluminum nanoparticles as thermal transfer surface an alternative the traditional aluminum.

The comparison clarified that the current heat exchanger at the ratio of the thermal content of the smallest to the largest (C_min_/C_max_ = 0.08) rises to the level of performance of the standard heat exchanger at the ratio (C_min_/C_max_ = 0). Further, it was found that the effectiveness of the current heat exchanger extremely reliant on the several factors such as: the extension of the vortices on the surface of the heat exchange which highest effectiveness, value observed at the relative length (L_S_/L = 0.42), and Taylor number which have had a positive effect on the effectiveness, since the effectiveness was increased significantly from (47%) to (50%), as Taylor number increased from zero to (1.76E4).

Further, It was confirmed that the effectiveness of the heat exchanger was effectively increased by using aluminum nanoparticles as an alternative the traditional aluminum. It was found that the maximum effectiveness of the exchanger improved from 47.7%, to 51.3%. To sum up, it could be concluded that the superior performance of the current heat exchanger could lead to the possibility of reducing the surface of the heat exchanger and thus reduce the size of a particular application.

## Declarations

### Author contribution statement

Walaa M. Hashim: Conceived and designed the experiments; Performed the experiments; Analyzed and interpreted the data; Contributed reagents, materials, analysis tools or data.

Hisham A. Hoshi: Performed the experiments; Analyzed and interpreted the data; Contributed reagents, materials, analysis tools or data.

Huda A. Al-Salihi: Conceived and designed the experiments; Analyzed and interpreted the data; Contributed reagents, materials, analysis tools or data; Wrote the paper.

### Funding statement

The authors received no funding from an external source.

### Competing interest statement

The authors declare no conflict of interest.

### Additional information

No additional information is available for this paper.
